# The effects of a topical gel containing chitosan, 0,2% chlorhexidine, allantoin and despanthenol on the wound healing process subsequent to impacted lower third molar extraction

**DOI:** 10.4317/medoral.21281

**Published:** 2016-07-31

**Authors:** Marta Madrazo-Jiménez, Ángela Rodríguez-Caballero, María-Ángeles Serrera-Figallo, Roberto Garrido-Serrano, Aida Gutiérrez-Corrales, José-Luis Gutiérrez-Pérez, Daniel Torres-Lagares

**Affiliations:** 1 Master in Oral Surgery. University of Seville; 2 Associate Professor of Dentistry in Handicapped Patients. University of Seville; 3 Professor of Oral Surgery. University of Seville

## Abstract

**Background:**

Despite efforts to prevent postoperative discomfort, there are still many immediate side effects associated with the surgical extraction of impacted lower third molars. Cicatrization is a physiological process through which the loss of integrity of oral mucosa is recovered and damaged tissues are repaired. Bexident Post (ISDIN, Spain) is a topical gel that contains chitosan, 0.2% chlorhexidine, allantoin and dexpanthenol. While this gel has many clinical indications, there are no published clinical trials evaluating its use in impacted mandibular third molar surgery. This study aims to clinically evaluate the efficacy of a gel containing chitosan, 0.2% chlorhexidine, allantoin and dexpanthenol on wound healing and reduction of postoperative side effects and complications after extraction of an impacted mandibular third molar.

**Material and Methods:**

A split-mouth design study was carried out on a total of 50 bilaterally and symmetrically impacted third molar extractions, which were randomly placed into either a control group (CG=25) or an experimental group (EG=25). Patients were all informed of the purpose of the study and provided written consent. All procedures were carried out by the same dental practitioner, in accordance with standard surgical protocol. A different dental practitioner, unaware of which treatment had been applied, provided follow-up care. The EG applied 10 ml of topical gel composed of chitosan, 0.2% chlorhexidine, allantoin and dexpanthenol to the surgical wound three times a day for 10 days, patients in the CG did not apply any gel.

**Results:**

The groups were homogeneous insofar as potentially confounding variables. No significant findings were found regarding postoperative swelling and pain. Neither of the groups displayed poor healing or infectious complications of the wound during the postoperative period. In all the recorded follow-ups (Day 7 *p*=0.001, and Day 14 *p*=0.01), the wound’s aesthetic appearance was better in the EG. Overall treatment tolerance was satisfactory and similar in both groups.

**Conclusions:**

The gel composed of chitosan, 0.2% chlorhexidine, allantoin and dexpanthenol did not aid in patients’ postoperative comfort; however, improved wound healing was observed.

**Key words:**Impacted lower third molar, postoperative wound healing, chitosan, chlorhexidine, allantoin, dexpanthenol, postoperative period.

## Introduction

Evolutionary reasons explain the lack of space jawbones have for proper eruption of a mandibular third molar, making it the most common dental impaction. Impacted third molars may lead to formation of cysts and tumors, resorption of adjacent teeth or pain of unknown origin. Preventive third molar extraction is recommended when risk of surgery is not higher than the benefit (1).

Recent studies reveal postoperative complications to be one of the most important factors in subjective self-assessment of patient satisfaction and perception of the surgical treatment received (2).

Despite efforts to prevent postoperative discomfort, immediate side effects of surgical extraction of impacted lower third molars include bleeding at the site of extraction, inflammation, pain, dysphagia and trismus (1). Other complications may arise, such as alveolar osteitis (0.3%-26%) (3), damage to the adjacent tooth (0.3%-0.4%) (4), hematoma (0.2%-5.8%) (3) and/or transient paresthesia, which can lead to permanent paresthesia (inferior alveolar nerve 0.35%; lingual nerve 0.69%) (3). Rarer complications include mandible fracture, the patient inhaling the tooth, and oro-antral communication (2).

Cicatrization is a physiological process through which the loss of integrity of oral mucosa is recovered and damaged tissues are repaired (5). The process begins with hemostasis and leads to three dynamic, interrelated phases: the inflammatory, proliferative and remodeling phases (5,6). These involve cell migration and transmigration, vasoconstriction, vasodilatation, angiogenesis, granulation tissue formation and extracellular matrix deposition.

Clearly, bacteria play a primary role in the processes of fibrinolysis, inflammation and poor healing, which appear as a result of infection at the extraction site (7). The most effective method of reducing risk of infection involves the use of topical or systemic agents that help eliminate oral bacteria (8).

Bexident Post is a topical gel commercialized by ISDIN labs (ISDIN, Spain) that contains chitosan, 0.2% chlorhexidine, allantoin and dexpanthenol. While evidence of clinical benefits exists for each of its components, there are no published clinical trials demonstrating its therapeutic benefits as a whole.

The present study seeks to clinically evaluate the efficacy of a gel containing chitosan, 0.2% chlorhexidine, allantoin and dexpanthenol in postoperative improvement after extraction of an impacted mandibular third molar. Observations were made of wound healing and reduction of postoperative side effects and/or complications (pain, inflammation, infectious complications, trismus, amount of analgesics required and patients’ overall perception of the procedure).

## Material and Methods

This prospective clinical split-mouth design study evaluated 25 patients from the dental school at the University of Seville in Spain. This study was approved by the Ethical Committee of Virgen del Rocío Hospital (Seville, Spain). The patients were treated between January 2012 and March 2013, requiring bilateral extraction of impacted lower third molars.

The selected patients were both male and female, aged 18 to 45, with symmetrically impacted lower third molars. They had a score between 5 and 7 on the Koerner Difficulty Index, exhibited no symptoms 15 days before surgery, had not taken any antibiotics or antiseptics 15 days before the surgery, and were highly eligible candidates for preventive extraction. Ineligible patients included those who did not understand the clinical procedures of the study, individuals allergic or intolerant to substances used in the study, patients receiving treatment affecting blood coagulation, patients under psychiatric treatment, pregnant or lactating women, women taking oral contraceptives, diabetic patients, individuals with periodontal disease or active infection, smokers of more than 10 cigarettes per day, patients with poor oral hygiene, and uncooperative patients, in addition to any patients whose surgical procedure lasted longer than 30 minutes.

A total of 50 extractions (25 patients) were randomly assigned to one of two groups: Experimental Group (EG) or Control Group (CG). The only difference between the two groups in terms of surgery protocol was that the EG applied 10 ml of topical gel composed of chitosan, 0.2% chlorhexidine, allantoin and dexpanthenol on the surgical wound three times a day for 10 days; patients in the CG did not apply any gel.

All patients were aware of the purpose of the present study and provided written consent of their participation. Patients’ complete medical history was reviewed, identifying their age, sex, hygiene practices, diet and toxic habits. The split-mouth study design guarantees a homogeneous distribution of confounding variables. Mouth opening and location of facial reference points were measured and identified prior to operation.

The same protocol was followed for all patients. The left impacted lower third molar was surgically removed with a follow-up period of 14 days, and after a washout period of 15 days the right impacted lower third molar was removed. The website www.random.org was used to assign every third molar to one of the two groups. They were assigned to a group after the first surgery, with the second (right) third molars automatically assigned to the other group. While the surgeon who carried out the surgeries and the patients were aware of which group they had been assigned, the practitioner who provided the follow-up evaluation was not.

The same surgeon performed all surgical procedures using the standard surgical technique described below. Additionally, patients received a follow-up evaluation from the same clinician (different from the surgeon).

The anesthetic used was 4% articaine with 1:100,000 adrenaline. In all patients, an envelope incision was made with a vertical extension mesial to the lower second molar, a mucoperiosteal flap was raised, ostectomy was performed using a No. 8 tungsten carbide bur on a surgical handpiece, and finally, a straight elevator was used to finish the extraction. If necessary, the molar was sectioned using a dental high-speed handpiece and fissure bur. The wound was irrigated with plenty of saline solution and wound edges were carefully sutured with simple interrupted stitches using 4.0 silk braided non-absorbable sutures (Laboratorio Aragó S. A.; Barcelona, Spain).

Patients were prescribed amoxicillin/clavulanate (875 mg/125 mg) and Ibuprofen (600 mg), to be taken every 8 hours for 7 days. They were also prescribed 500 mg of paracetamol (acetaminophen) for use as a rescue analgesic as need. The only difference between the two groups in terms of surgery protocol was that the EG applied 10 ml of topical gel composed of chitosan, 0.2% chlorhexidine, allantoin and dexpanthenol on the surgical wound three times a day for 10 days; patients in the CG did not apply any gel. Treatment compliance is certified with the collection of the excess gel.

**Figure 1:**
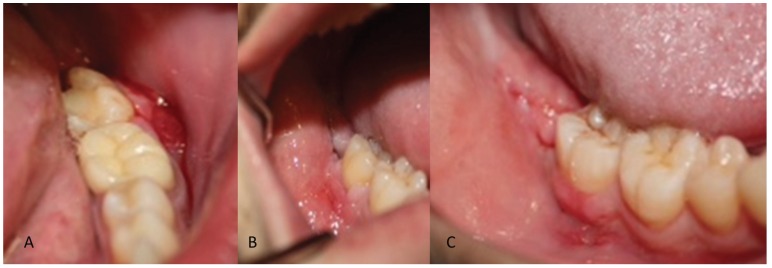
Acceptable wound healing. Note the dehiscence in the horizontal incision (A) and the vertical incision discharge (B). Good wound healing. Note the lack of dehiscence, among other aspects (C).

The following variables were measured: wound consistency and appearance were categorized as good, acceptable or bad ( Table 1, Fig. 1). Preoperative and postoperative pain levels were recorded for 14 days following the surgery (beginning 6 hours after surgery) using a visual analog scale from 0 to 10 (with 0 being no pain at all and 10 the worst possible pain). Level of inflammation was recorded both 3 and 8 days post-surgery by measuring in millimeters the following facial points, which represented the overall size of the patient’s face at any given time: mandibular angle, tragus, lateral canthus of the eye, the base of the nasal ala, oral commissures and pogonion. These values were then used to make the following calculation: Postoperative measurement (mm) - preoperative measurement (mm) = amount of inflammation (mm). Level of trismus was determined by measuring the interincisal distance 3 and 8 days post-surgery using a scaled ruler. Patients’ overall treatment tolerance was recorded on a visual analog scale from 1 to 5, with 1 being a good level of tolerance to a compound and 5 a very bad one. Total surgery time was also recorded, with any surgery procedures that lasted longer than 30 minutes being excluded.

**Table 1:**
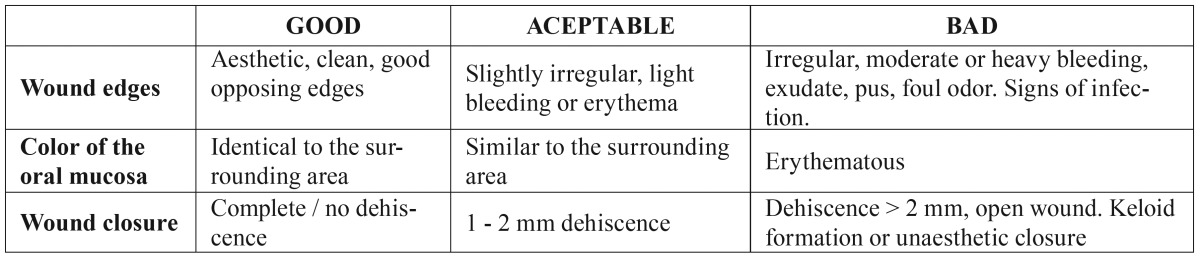
Criteria used to assess the wound’s healing process.

Statistical analysis was carried out using the IBM SPSS Statistics v.23 software (IBM, United States). Descriptive parameters (frequency, mean and standard deviation) of the recorded variables were calculated. The difference between both groups was assessed using the independent-samples t-test (*p*<0.05) and the Chi-square test.

## Results

The mean age of patients was 26.47 years ± 6.74 in both groups. Both groups had an equal gender distribution (15 men, 60% and 10 women, 40%), neither group had any prior medical history or allergies, and there were no differences found in hygiene practices, diet or toxic habits.

The average time of extraction was 14.34 minutes ± 3.45 in the EG and 13.76 ± 4.32 minutes in the CG. The difficulty index was 6.05 ± 0.65 in the EG and 6.01 ± 0.54 in the CG. Initial limitation of mouth opening was similar in both groups (EG=52.56 mm ± 6.9; CG=52.1 mm ± 6.94). No significant differences were found. Initial facial measurements were 19.86 mm ± 1.27 in the EG and 19.61 mm ± 1.10 in the CG ( Table 2, *p*>0.05).

**Table 2:**
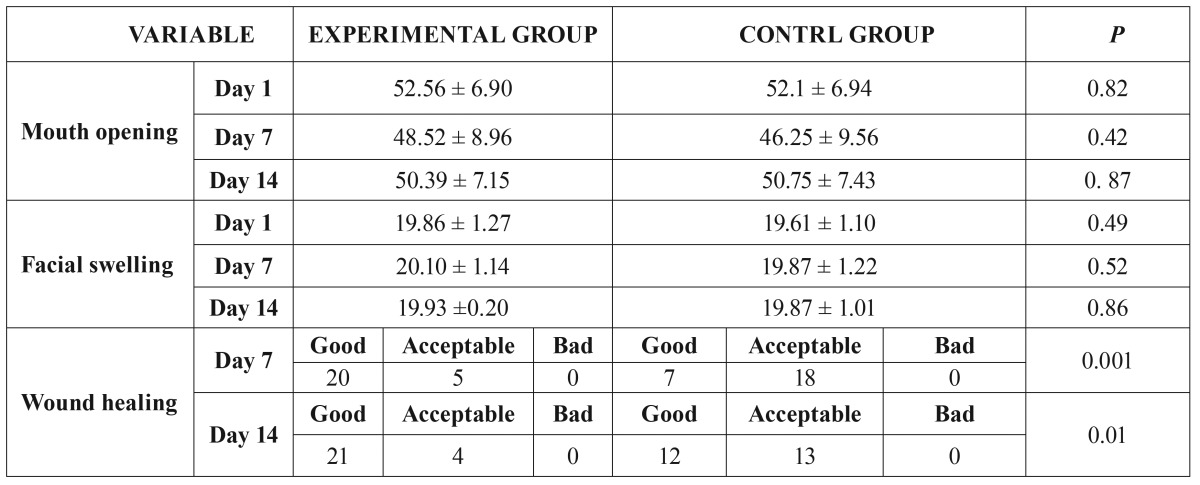
Comparative table of mouth opening, facial measurements and wound healing in the experimental and control groups.

**Figure 2:**
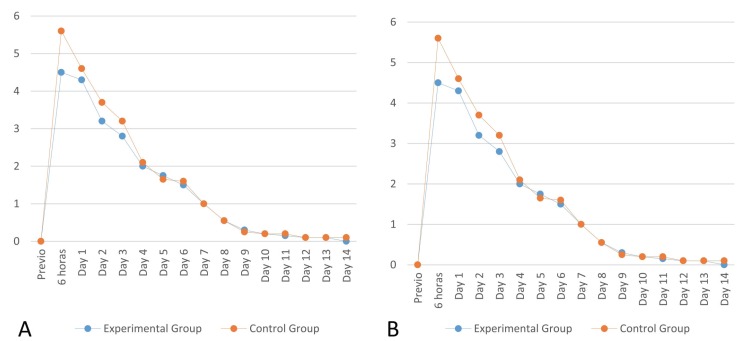
Comparative chart of pain (A) and swelling (B) levels of both groups during the postoperative period.

Throughout the postoperative period, no significant difference in swelling and pain was observed between the two groups. There was a slight improvement in swelling of the EG during the first days after the surgery, but not enough to be deemed significant (Fig. 2). No significant differences were found in limitation of mouth opening or facial swelling (level of inflammation) during the study’s follow-up evaluations ( Table 2). The statistical power to identify a change in the mouth opening of 5 mm (50 to 55 mm for example), with a standard deviation of 7 mm and 25 patients per group was 81.1%. The statistical power to identify a change of 1.5 cm in facial swelling (for example, from 20 cm to 21.5 cm), with a standard deviation of 1.5 and 25 patients per group was 97.1%.

**Figure 3:**
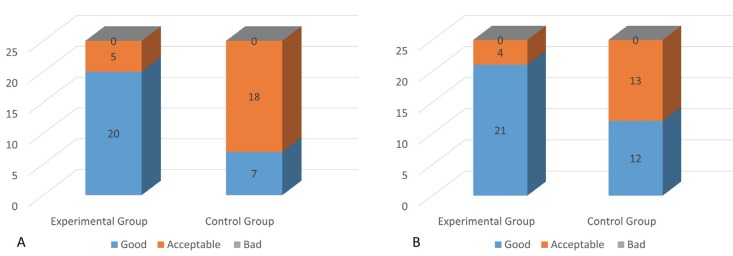
Wound healing of both groups on day 7 (A) and day 14 (B) of the study.

Wound appearance and consistency were better in the EG, both on day 7 (good healing, 20/25 (80%) vs. 7/25 (28%), *p*=0.001) and day 14 (good healing, 21/25 (84%) vs. 12/25 (48%), *p*=0.01) of follow-up. Neither of the groups displayed poor healing or infectious complications of the wound throughout the postoperative period ( Table 2, Fig. 3). Overall treatment tolerance was satisfactory and similar in both groups (4.32 ± 0.54 *p*>0.5).

## Discussion

A total of 50 impacted lower third molar extractions were evaluated. They were divided into two groups: 25 in the Control Group and 25 in the Experimental Group. Both groups displayed similar characteristics, an advantage of the split-mouth study design. No significant differences were seen in the surgical difficulty index or the time it took to complete the surgery. The same surgeon performed all surgeries included in this study, and the same clinician (different from the surgeon) carried out the follow-up evaluation of all patients. No allergic reactions or infectious complications were observed after the surgery. Wound healing was deemed either good or acceptable in all cases; no wound was categorized negatively. However, some significant differences were seen in improved appearance and wound closure in the group treated with the topical gel under study (EG).

The clinical indications of the topical gel containing chitosan, 0.2% chlorhexidine, allantoin and dexpanthenol, (Bexident Post, ISDIN, Spain) are well known; however, there are no published clinical trials demonstrating its therapeutic effectiveness as a whole. Nevertheless, there are various different publications on the therapeutic uses of each component.

Allantoin (5-ureidohydantoin) has numerous widely cited pharmacological uses, including wound healing (9), anti-irritation, hydration and regeneration of tissue (9) and cell proliferation (10), as well as having analgesic (11), keratolytic (12) and epithelially stimulating effects (11). Although allantoin has been used for over 70 years, especially as a wound healer, not enough clinical trials exist and so its mechanism of action remains unknown (13).

Chlorhexidine (CHX) is a bis-guanide antiseptic that displays a wide range of antimicrobial activity, in addition to its safety, effectiveness, substantivity and low toxicity (5). CHX now comes in many different forms, including mouthwashes, bioadhesive gels, dental varnishes, and chips (1,7).

Chitosan is a naturally non-toxic, bioactive polysaccharide that lends itself to a wide range of biomedical applications due to its high compatibility, biodegradation and cationic nature, as opposed to other polysaccharides with a neutral or negative charge (14). The properties and many uses of chitosan and its derivatives have been noted in various different studies (14-17). Chitosan is known for its antibacterial, antifungal, antioxidant, anti-diabetic and anti-inflammatory uses, in addition to its cholesterol-lowering properties.

Within the skin, dexpanthenol (provitamin B5) is metabolized into pantothenic acid (vitamin B5), which is essential for the normal functioning of epithelial cells, especially during the first phase of epithelial regeneration (within the first four days) (18,19). Dexpanthenol has been used in dermatological applications to treat wound healing disorders, dermatosis, scratches, extensive burns or skin transplants (18-20). For decades, dermatologists have routinely used dexpanthenol for its anti-inflammatory effects and its role in epithelial protection (18).

All the information collected shows that this is the first published clinical trial to evaluate a combination of 0.2% chlorhexidine, allantoin, chitosan and dexpanthenol as used to aid in postoperative healing (Bexident Post, ISDIN, Spain). The most satisfactory results were observed in wound healing after extraction of an impacted lower third molar. Although there are no similar studies with which to compare the present study, its results can still be compared with those of clinical trials evaluating the different compounds mentioned, as well as with other studies that also seek to improve postoperative healing after oral surgery. This information is further supported by already published proof of each compound’s efficacy.

Use of 0.2% chlorhexidine mouthwash and bioadhesive gel before and after surgery appears to play an important role in the improvement of preoperative oral hygiene conditions, which improves the wound healing process. However, these results are controversial (21,22). Similarly, topical application of minocycline or lincomicin during and after surgery yields positive results and improves the patient’s quality of life after surgery (22).

A recent study compared the effects of a gel composed of onion extract, sodium heparin and allantoin on the wound healing process. Results found significant improvement in the quality of cicatrization, as well as reduced formation of keloids and/or hypertrophic scars (23).

Another clinical trial was found in which a 3 mm incision was made on the palate of 125 rats. There were 4 experimental groups and 1 control group; each of the five groups received 0.2% chlorhexidine gel, hyaluronic acid gel, 0.5% allantoin gel, placebo gel or nothing, respectively. None of the tested agents were shown to adversely affect wound healing. The best results were obtained in the groups in which 0.2% chlorhexidine and hyaluronic acid were applied (24).

Chitosan’s effects on wound healing after oral surgery are best seen in a study conducted on the effects of local application of glutathione (GHS) and chitosan on oxidative processes and the histological changes that occur in the oral mucosa of rats during the wound healing process. According to the histological findings, the group treated with chitosan showed better results than the other groups. The study demonstrated that local application of chitosan on intraoral wounds, whether by itself or mixed with glutathione, can aid in the wound healing processes of soft tissues (25).

Finally, one study examined dexpanthenol’s effectiveness in pediatric pain management after frenectomy and throughout the wound healing process. The group that took dexpanthenol suffered less pain than the placebo group, and wound healing was accelerated (26).

Other research includes the postoperative use of hyaluronic acid gel, which has anti-inflammatory effects, although oxidative stress and clinical results are similar by one week after surgery (27); platelet-rich plasma (PRP) and platelet-rich fibrin (PRF), which proved very beneficial in the cicatrization of soft tissue and helped speed up the wound’s healing process (28); and photodynamic and ozone therapy, which reduce bleeding and exudate, decrease the wound’s temperature and improve the patient’s quality of life during the postoperative period (29).

The relationship between psychological and physiological variables in predicting the likelihood of good postoperative recovery has also been studied. Results show that psychological factors such as stress, high need for a sense of control or overly vigilant attitude may influence the postoperative process, regardless of the effects of any physical trauma resulting from the surgery (30).

Apart from its limited sample size, the main limitation of the present study is that it is not a double-blind trial.

Results showed a significant improvement in healing of the surgical wound when a gel comprised of chitosan, 0.2% chlorhexidine, allantoin and dexpanthenol (Bexident Post, ISDIN, Spain) was applied after extraction of impacted third molars. Patients can benefit from an effective, simple and accessible measure that increases the likelihood of a favorable outcome in the closure and healing of wounds after extraction of an impacted lower third molar. However, the gel does not help prevent swelling or pain, both of which can be alleviated with other medication.

In conclusion, the collected data highlight a need for further research to evaluate the gel’s effect on a larger patient sample size, comparing it with a placebo group and/or current methods of postoperative treatment.
